# SPOP suppresses prostate cancer through regulation of CYCLIN E1 stability

**DOI:** 10.1038/s41418-018-0198-0

**Published:** 2018-09-20

**Authors:** Lin-Gao Ju, Yuan Zhu, Qiao-Yun Long, Xue-Jing Li, Xiang Lin, Shan-Bo Tang, Lei Yin, Yu Xiao, Xing-Huan Wang, Lianyun Li, Lei Zhang, Min Wu

**Affiliations:** 1 Hubei Key Laboratory of Cell Homeostasis, Hubei Key Laboratory of Developmentally Originated Disease, College of Life Sciences, Wuhan University, Wuhan, Hubei 430072 China; 2 Department of Urology, Zhongnan Hospital, Wuhan University, Wuhan, Hubei 430072 China; 3 State Key Laboratory of Cell Biology, CAS Center for Excellence in Molecular Cell Science, Innovation Center for Cell Signaling Network, Shanghai Institute of Biochemistry and Cell Biology, Chinese Academy of Sciences, University of Chinese Academy of Sciences, Shanghai, 200031 China

**Keywords:** Tumour-suppressor proteins, Tumour-suppressor proteins

## Abstract

SPOP is one of the important subunits for CUL3/SPOP/RBX1 complex tightly connected with tumorigenesis. However, its exact roles in different cancers remain debatable. Here, we identify CYCLIN E1, as a novel substrate for SPOP. SPOP directly interacts with CYCLIN E1 and specific regulates its stability in prostate cancer cell lines. SPOP/CUL3/RBX1 complex regulates CYCLIN E1 stability through poly-ubiquitination. CDK2 competes with SPOP for CYCLIN E1 interaction, suggesting that SPOP probably regulates the stability of CDK2-free CYCLIN E1. CYCLIN E1 expression rescued proliferation, migration, and tumor formation of prostate cancer cell suppressed by SPOP. Furthermore, we found SPOP selectively regulates the substrates’ stability and signaling pathways in prostate cancer and CCRC cell lines, suggesting that complicated mechanisms exist for SPOP to regulate substrate specificity. Altogether, we have revealed a novel mechanism for SPOP in suppressing prostate cancer and provided evidence to show SPOP has dual functions in prostate cancer and CCRC.

## Introduction

SPOP was initially identified as a nuclear protein that exhibits a speckled localization pattern [1]. Nuclear speckles are considered as an important structure for RNA editing and maturation, where SPOP is thought to be involved in RNA processing and transcription regulation [2]. Recent studies have established that SPOP forms an ubiquitin E3 ligase complex with cullin 3 (CUL3) and ring-box 1 (ROC1/RBX1), which poly-ubiquitinates substrates with K48 ubiquitin chain and promotes protein degradation via proteasome [3]. Sequence alignment and structure study revealed a peptide sequence, φ-π-S-S/T-S/T, as SPOP-binding consensus (SBC) motif [3]. A bunch of substrates for SPOP have been identified, including death-domain associated protein (DAXX), macro H2A, androgen receptor (AR), nuclear receptor co-activator 3 (NCOA3), phosphatase and tensin homolog (PTEN), ETS transcription factor (ERG), DEK proto-oncogene (DEK), cell division cycle 20 (CDC20), inverted formin, FH2 and WH2 domain-containing (INF2), bromodomain containing 4 (BRD4) and SET domain-containing 2 (SETD2) in mammals, as well as cubitus interruptus (Ci) in *Drosophila* [4–19]. Most of these substrates are localized in nucleus, further suggesting SPOP as a transcription regulator.

Extensive genomic studies identified SPOP as a tumor-suppressor in prostate cancer (PCa) tissues [20, 21]. Multiple studies have illustrated that SPOP exerts its tumor suppressive function through degradation of oncogenic substrates in PCa [21]. Among them, NCOA3/SRC3 is a co-activator for androgen receptor (AR) and promotes PCa tumorigenesis [7, 19]; DEK acts as a proto-oncogene involved in mRNA processing and DNA replication [11]; ERG is one member of ETS family of transcription factor, and considered as one marker for prostate cancer [10]. SPOP mutation has recently been proved to be the major cause in PCa exhibiting resistance to BET inhibitor [15–18]. Interestingly, the nonsense mutation of SPOP is seldom found in PCa, suggesting SPOP mutants may play more complicated roles in tumorigenesis [20].

SPOP was also identified as an oncogene in clear cell renal carcinoma (CCRC) [22]. SPOP is overexpressed in more than 90% CCRC tissues [23]. It was reported that SPOP promotes CCRC through degradation of PTEN, a well-known tumor-suppressor [9]. Our group have recently identified SETD2 as one of SPOP substrates, which is the major histone H3K36me3 methyltransferase in mammal and a tumor-suppressor in many cancers [12, 24]. Moreover, one poly-peptide inhibitor of SPOP, has been shown to be effective in treating CCRC in an animal model [25].

Thus, a debate is currently existing in the field about SPOP’s function in tumorigenesis [21, 22]. Whether is SPOP an oncogene or tumor-suppressor? Or does it play different roles in the context dependent manner? It is necessary to further explore the underlying molecular mechanisms and compare its effect in two cancers side by side.

In the present study, we have revealed a new mechanism for SPOP in repressing PCa and identified CYCLIN E1, an oncogene and important cyclin involved in G1/S transition, as a new SPOP substrate. We show here SPOP interacts with CYCLIN E1, selectively regulates its stability in prostate and bladder cancer cell lines, and regulates PCa tumorigenesis through CYCLIN E1 degradation. Furthermore, we show here SPOP has substrate specificity and regulates distinguished transcription programs in PCa and CCRC cell lines, suggesting complicated mechanisms exit for SPOP substrate selectivity in different cancers.

## Results

### The interaction between exogenous expressed SPOP and CYCLIN E1

**Fig. 1:**
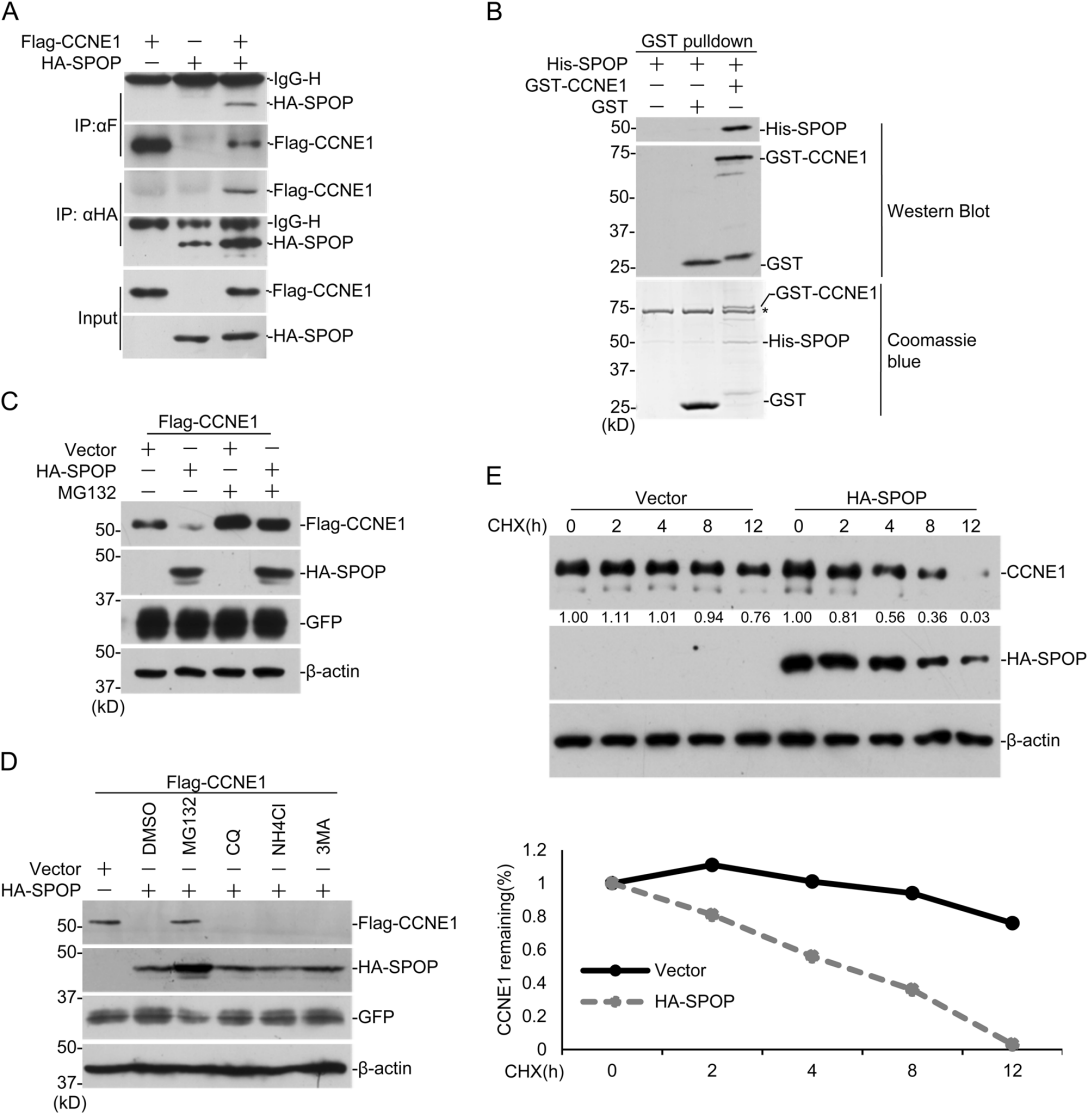
SPOP regulates the stability of exogenous expressed CYCLIN E1. **a** HEK293 cells were transfected with indicated plasmids for 24 h followed by treatment with 10 μM MG132 for 12 h, and co-IP was performed with anti-Flag or anti-HA antibodies. **b** His-SPOP and GST-CCNE1 were expressed in bacteria and affinity purified. GST-pulldown assay was performed to study the direct interactions between CYCLIN E1 and SPOP. **c** HEK293 cells were transfected with indicated plasmids. Twenty-four hours after transfection, cells were treated with 10 μM MG132 for 12 h and harvested for WB. **d** HEK293 cells were transfected with indicated plasmids. After 24 h, cells were treated with DMSO, MG132 (10 μM for 12 h), CQ (20 μM for 9 h), NH_4_Cl (20 mM for 9 h), 3-MA (5 mM for 9 h), and harvested for WB. **e** DU145 cells were transfected with the indicated plasmids and treated with 100 μg/ml cycloheximide (CHX) 24 h later. Cells were then harvested at various time points for WB. CYCLIN E1 protein abundance was quantified by ImageJ and plotted as indicated. *means *p*-value < 0.05; **means *p*-value < 0.01

When exploring the underlying mechanisms for SPOP-regulated tumorigenesis, we found that the exogenous expressed SPOP and CYCLIN E1 interact with each other in HEK293 cells (Fig. 1a). We further expressed and purified His-tagged SPOP and GST-tagged CCEN1 in bacteria and performed GST pulldown assay, which indicates that SPOP and CYCLIN E1 can interact directly (Fig. 1b). To map the interaction domains between the two proteins, a series of truncated forms of CYCLIN E1 and SPOP were generated (Sup. Fig. 1A, B). The immunoprecipitation assays indicated that the N-terminal MATH domain of SPOP interacts with CYCLIN E1, and the cyclin domain in middle of CYCLIN E1 interacts with SPOP (Sup. Fig. 1C, D). Since SPOP is a ubiquitin E3 ligase, we speculate that CYCLIN E1 may be one of the substrates for SPOP.

### SPOP regulates CYCLIN E1 stability in a proteasome-dependent manner

To study whether SPOP regulates CYCLIN E1 stability, Flag-tagged CYCLIN E1 was expressed in HEK293 and its protein level was dramatically decreased with HA-SPOP (Fig. 1c). MG132 treatment successfully rescued it, suggesting CYCLIN E1 degradation by SPOP is proteasome dependent (Fig. 1c). We further tested the effects of NH_4_Cl, chloroquine (CQ) and 3-MA, inhibitors for lysosome and autophagy, respectively. Only MG132 inhibited the effect of SPOP on CYCLIN E1 stability, further suggesting the process is dependent on proteasome (Fig. 1d). Then cells were treated with cycloheximide (CHX), and the decay rate of CYCLIN E1 was studied. The result indicated that the expression of *SPOP* significantly accelerates the degradation of endogenous CYCLIN E1 (Fig. 1e). Consistent with above interaction studies, CCNE1-NC, which lacks the cyclin domain, remained stable with *SPOP* expression (Sup. Fig. 1E). And only full length SPOP degraded CYCLIN E1, suggesting both MATH and BTB domains are required (Sup. Fig. 1f). These suggest exogenous expressed SPOP promotes CYCLIN E1 degradation through interaction between MATH domain in SPOP and cyclin domain in CYCLIN E1.

### Regulation of endogenous CYCLIN E1 stability by SPOP specifically in prostate and bladder cancer cells

**Fig. 2:**
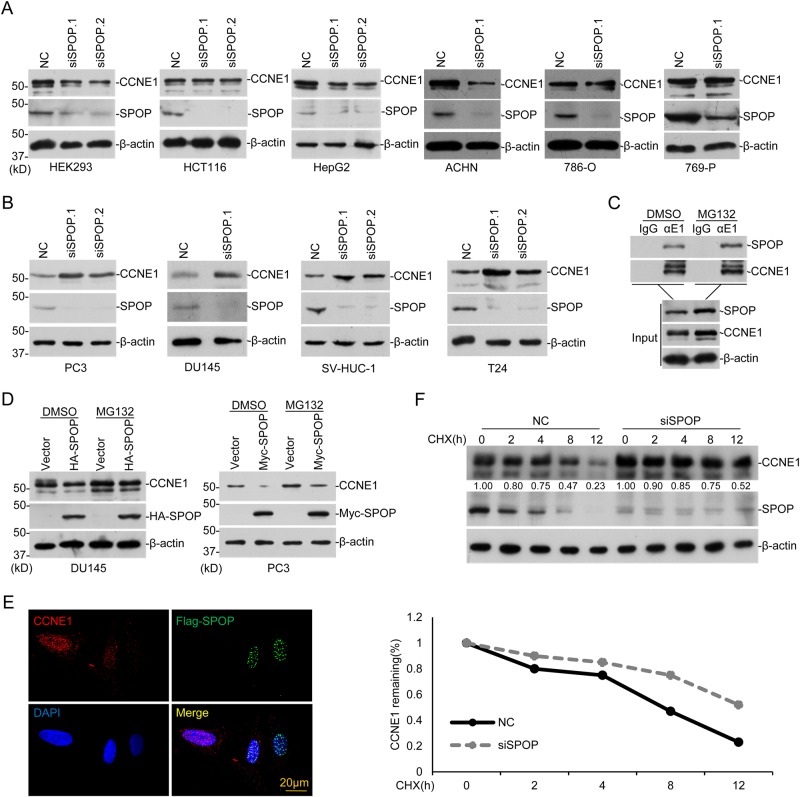
SPOP regulates endogenous CYCLIN E1 specific in prostate and bladder cancer cell lines. **a**, **b** The indicated cell lines were transfected with control or *SPOP*-specific siRNAs. After 72 h, cells were harvested for WB. **c** After treatment with DMSO or MG132 (10 mM) for 12 h, DU145 lysates were prepared for co-IP with CYCLIN E1 antibody. **d** DU145 and PC3 cells were transfected with HA- or Myc-SPOP plasmid. After 24 h, cells were treated with DMSO or MG132 (10 mM) for 12 h and endogenous CYCLIN E1 level was assayed with WB. **e** Flag-SPOP (green) was expressed in PC3 cells and endogenous CYCLIN E1 (red) was assayed by immunofluorescent staining. **f** *SPOP* was knocked down in DU145 cells, and 48 h later cells were treated with 100 μg/ml cycloheximide (CHX), and harvested at various time points for WB. CYCLIN E1 protein abundance was quantified by ImageJ and plotted as indicated. *means *p*-value < 0.05

The above data suggest CYCLIN E1 may serve as a new substrate for SPOP, then we started to investigate whether it occurs at the endogenous level. The endogenous CYCLIN E1, SPOP, and CDK2 levels in some cell lines were studied (Sup. Fig. S2A) and totally 15 cell lines were screened. It is interesting that *SPOP* knockdown caused increase of CYCLIN E1 only in four cell lines, DU145 and PC3, two prostate cancer cell lines, SV-HUC-1 and T24, two bladder cell lines, but not in others, including four kidney cell lines (ACHN, 786-O, 769-P, and HEK293), one colon cancer cell line (HCT116), two liver cell lines (HepG2 and HL7702), one bone osteosarcoma cell line (U2OS), one cervical cancer cell line (HeLa), one bladder cancer cell lines (UM-UC-3) and one prostate cancer cell line (22RV1) (Fig. 2a, b and Sup. Fig. S2B–F). Quantitative reverse transcription PCR (RT-PCR) showed that *SPOP* knockdown did not affect *CCNE1* mRNA level in DU145 and 769-P (Sup. Fig. S2G, H). These together suggest that the regulation of CYCLIN E1 stability by SPOP is cell line specific, and may be selectively occurs in some types of prostate and bladder cancer cell lines.

In DU145, we successfully verified the interaction between endogenous CYCLIN E1 and SPOP with co-immunoprecipitation (Fig. 2c). Overexpression of HA-SPOP decreased the endogenous CYCLIN E1 in DU145 and PC3 (Fig. 2d). Immunofluorescent staining further showed that expression of Flag-SPOP in DU145 reduced CYCLIN E1 level in the cell, in comparison with un-transfected cells (Fig. 2e). With CHX treatment, SPOP knockdown also slowed down CYCLIN E1 degradation in DU145 (Fig. 2f). In DU145, wild-type SPOP downregulates CYCLIN E1 but not the tested SPOP mutants (Sup. Fig. 2I). All these indicate that SPOP regulates CYCLIN E1 stability in prostate and bladder cancer cell lines.

### Poly-ubiquitination of CYCLIN E1 by SPOP/CUL3/RBX1 complex

**Fig. 3:**
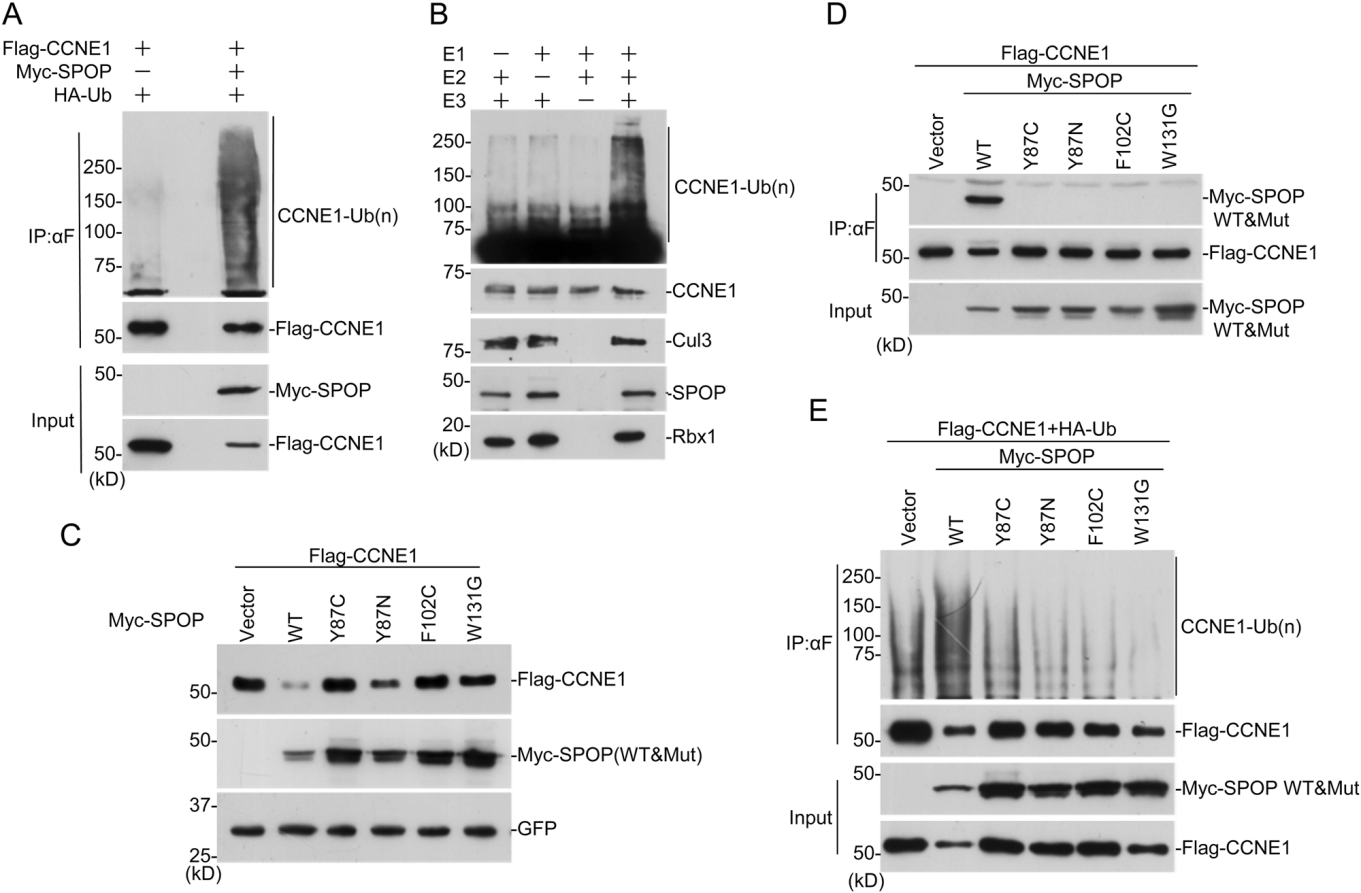
SPOP/CUL3/RBX1 complex poly-ubiquitinates CYCLIN E1. **a** HEK293 cells were transfected with indicated plasmids for 24 h followed by MG132 (10 mM) treatment for 12 h. Immunoprecipitated Flag-CCNE1 were analyzed for ubiquitination with anti-HA WB. **b** SPOP/CUL3/RBX1 complex was expressed in insect cells and affinity purified. In vitro ubiquitination assay was performed together with bacteria-expressed E1, E2, and GST-CCNE1. **c** Flag-CCNE1 was expressed in HEK293 cells with SPOP wild-type or mutants. Twenty-four hours after transfection, cells were harvested for WB. **d** HEK293 cells were transfected with indicated plasmids. Co-immunoprecipitation was performed to study CYCLIN E1-SPOP interaction after treatment with 10 μM MG132 for 12 h. **e** HEK293 cells were transfected with indicated plasmids and treated with 10 μM MG132 for 12 h. Ubiquitination assay was performed to study the effect of SPOP mutants on CYCLIN E1 ubiquitination

To test whether CYCLIN E1 is one of the substrates for SPOP, in vivo and in vitro ubiquitination assays were performed. SPOP expression greatly increased the poly-ubiquitination of CYCLIN E1 in HEK293 cells (Fig. 3a). Then SPOP/CUL3/RBX1 complex was expressed and purified with baculovirus system in insect cells. CYCLIN E1 was expressed in bacteria and purified with affinity columns. The purified SPOP/CUL3/RBX1 complex successfully poly-ubiquitinated CYCLIN E1 in vitro, with the presence of E1 and E2 (UBCH5C) (Fig. 3b). These demonstrated that SPOP/CUL3/RBX1 complex can directly poly-ubiquitinates CYCLIN E1.

To examine the effect of SPOP mutants in prostate cancer on CYCLIN E1, four constructs containing most frequent SPOP mutations were generated. When CYCLIN E1 was co-expressed with these mutants, it was much more stable than that with wild-type SPOP (Fig. 3c). Co-immunoprecipitation assay showed that only wild-type SPOP interacts with CYCLIN E1, but not the mutants (Fig. 3d). Ubiquitination assay indicated that SPOP mutants caused less poly-ubiquitination chain on CYCLIN E1 than wild type (Fig. 3e). These suggest that SPOP mutants in prostate cancer have less activity to promote CYCLIN E1 degradation.

### CDK2 inhibits CYCLIN E1 poly-ubiquitination by SPOP

**Fig. 4:**
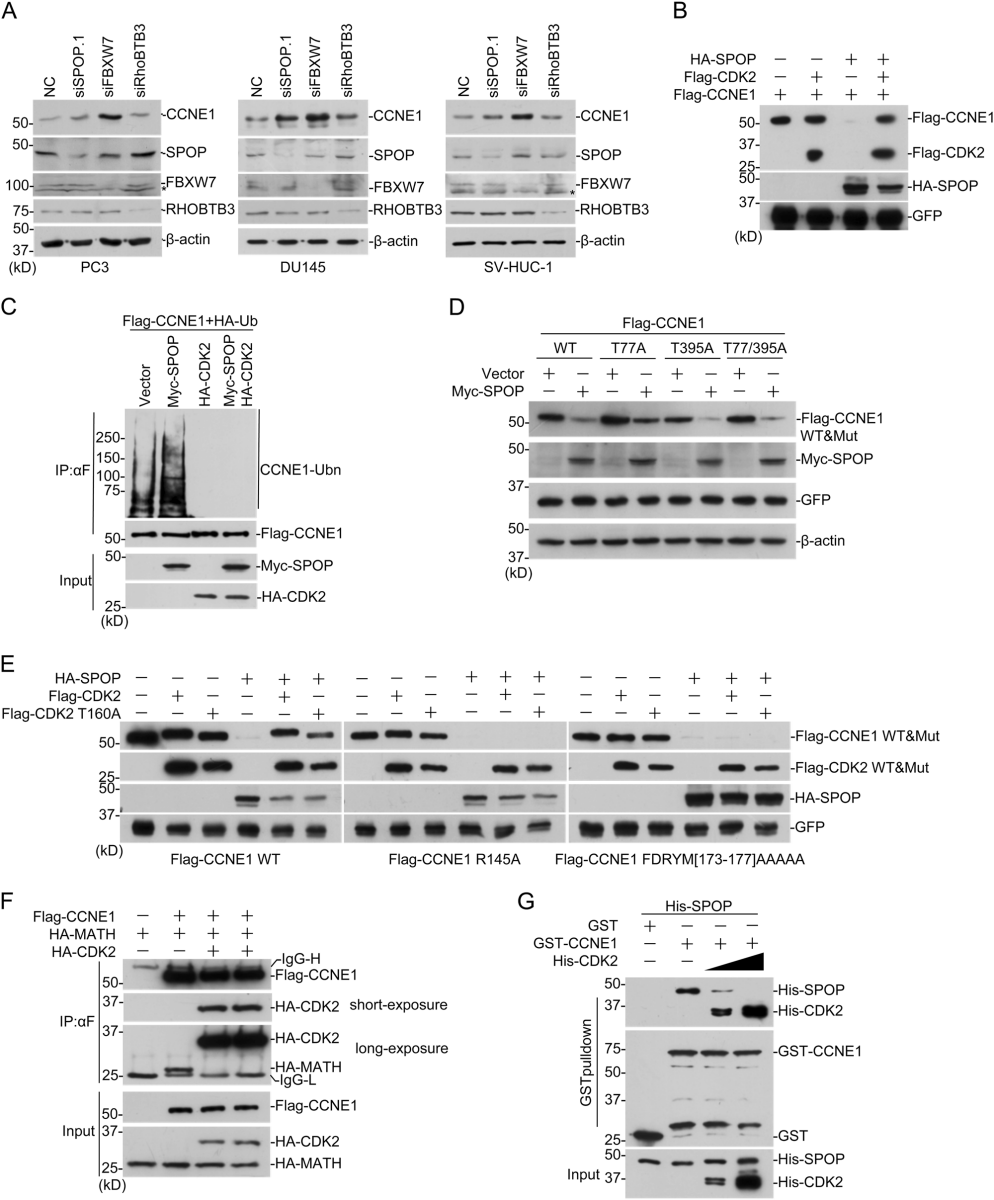
CDK2 inhibits CYCLIN E1 degradation promoted by SPOP. **a** PC3, DU145, SV-HUC-1 cells were transfected with *SPOP/FBXW7/RhoBTB3* siRNAs. Seventy-two hours after transfection, cells were harvested for WB. **b**, **c** HEK293 cells were transfected with indicated plasmids. Twenty-four hours later, cells were harvested and Flag-CCNE1 protein level (**b**) and ubiquitination (**c**) were studied. **d** *CCNE1* plasmids were constructed with mutation of CDK2 phosphorylation sites. Then CYCLIN E1 mutants were expressed in HEK293 with or without SPOP and western blotting was performed to study CYCLIN E1 protein level. **e** CYCLIN E1 mutants abolishing CDK2 interaction were generated and co-expressed with CDK2 wild-type or kinase dead mutant in HEK293. Western blotting was performed to study CYCLIN E1 protein stability. **f** Cells were transfected with indicated plasmids and the interaction between Flag-CCNE1 and HA-SPOP (MATH domain) was studied with co-IP. **g** GST-pulldown was performed with bacteria-expressed proteins. The addition of His-CDK2 inhibits the interaction between GST-CCNE1 and His-SPOP

Previously, two ubiquitin E3 ligases for CYCLIN E1 were reported, FBXW7 and RhoBTB3 [26–28]. To compare the effects of three E3 ligases, we knocked down each of them with siRNAs. It seemed that all three E3 ligases regulate CYCLIN E1 in these cells, while FBXW7 seemed to be most powerful and RHOBTB3 only showed a slight effect (Fig. 4a). CYCLIN E1 phosphorylation by CDK2 is required for degradation by FBXW7 [26, 29]. To investigate the role of CDK2 in SPOP-dependent CYCLIN E1 degradation, Flag-CCEN1 and HA-SPOP were expressed in cells with or without Flag-CDK2. The result suggest that CDK2 expression inhibited CYCLIN E1 degradation by SPOP (Fig. 4b). Ubiquitination assay showed that CDK2 expression also inhibited CYCLIN E1 poly-ubiquitination by SPOP (Fig. 4c). We generated three CYCLIN E1 mutants, T77A, T395A, and T77/395A, whose phosphorylation sites by CDK2 and GSK3 were mutated to alanine. According to the previous reports, they should resist the degradation promoted by FBXW7 (Sup. Fig. S2J) [30]. Interestingly, all of the three mutants can still be degraded by SPOP (Fig. 4d), suggesting that distinguished regulatory mechanisms exist for FBXW7- and SPOP-modulated CYCLIN E1 degradation.

### CDK2 competes with SPOP for CYCLIN E1 binding

To explore the underlying mechanism how CDK2 inhibits SPOP-dependent CYCLIN E1 ubiquitination, we used a CDK2 kinase dead mutant, T160A, and found it can still inhibit CYCLIN E1 degradation by SPOP as wild-type (Fig. 4e). Meanwhile, two CYCLIN E1 mutants, R145A and 5A^173-177^, which both abolish the interaction with CDK2 [30, 31], were degraded by SPOP in the presence of CDK2 (Fig. 4e). These suggest that interaction with CDK2, but not CDK2 kinase activity, is required for inhibition. When CDK2 was expressed in the cell, the interaction between MATH domain in SPOP and CYCLIN E1 was greatly impaired (Fig. 4f). To further study the effect, we performed GST-pulldown assay with bacteria-expressed proteins, and found that addition of His-CDK2 repressed the interaction between GST-CCNE1 with His-SPOP (Fig. 4g). These suggest that CDK2 competes with SPOP for CYCLIN E1 binding.

### SPOP deficiency impairs S phase progression of synchronized cells

**Fig. 5:**
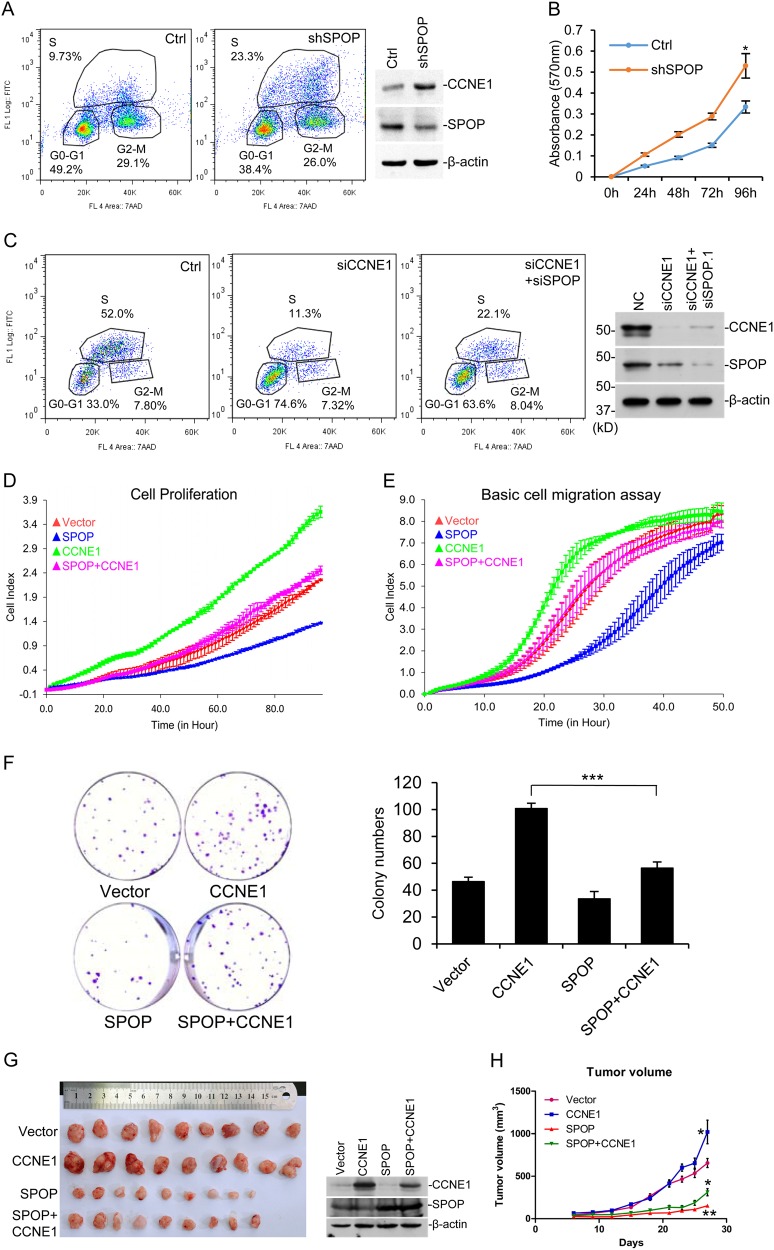
SPOP inhibits proliferation and migration of prostate cancer cell via CYCLIN E1. **a** *SPOP* stable knockdown cell line in DU145 was made with retroviral infection system. Cells were synchronized with double-thymidine block, and 8 h after release cells were pulse-labeled with 10 μM BrdU for 30 min and assayed with flow cytometry **b** Cell proliferation of *SPOP* knockdown cells in **a**, assayed with MTT. *means *p*-value < 0.05. **c** *SPOP* or *CCNE1* was transiently knocked down with siRNAs in DU145 cells as indicated. BrdU-incorporation assay was carried out 4 h after release from synchronization. **d**, **e** SPOP and CYCLIN E1 stable expressed DU145 cells were generated with lentivirus expressing system. The real-time cell proliferation (**d**) and migration (**e**) were measured with RTCA assay according to the manufacturer’s protocol. **f** Plate colony formation assay of CYCLIN E1 and SPOP stable expressed DU145 cells. Colony numbers was counted and plotted as indicated. ***means *p-*value = 0.0005. **g**, **h** Xenograft experiments of SPOP and CYCLIN E1 stable expressed DU145 cells. In all, 5 × 10^6^ cells were injected subcutaneously into the nude mice and tumors were harvested 27 days later. Tumors were pictured (**g**) and tumor volumes were shown as mean ± SD, *n* = 9 (**h**). One tumor from each group was random picked and assayed with western blotting to confirm they are from original cell lines (**g**, right). * means *p* < 0.05

CYCLIN E1 is an important cyclin during S phase progression. We synchronized cells with double-thymidine block and then released them to study whether SPOP regulates S phase progression through CYCLIN E1. However, we only got *SPOP* stable knockdown cell line, but not that for *CCNE1*, probably due to the cell cycle defect without CYCLIN E1. The flow cytometry results indicated SPOP deficiency increased S phase cells after release (Fig. 5a). MTT assay showed that SPOP deficiency increased cell proliferation (Fig. 5b); and the colony number formed by DU145 cells (Sup. Fig. S3A). The transient knockdown of *CCNE1* with siRNA led to reduction of S phase as expected; and co-knockdown of both genes with siRNA partially rescued the *CCNE1* siRNA phenotype (Fig. 5c).

### SPOP represses tumor phenotypes in a CYCLIN E1-dependent manner

Since we could not get *CCNE1* stable knockdown or knockout cell line, we stably expressed CYCLIN E1 and SPOP in DU145, separately or together (Sup. Fig. S3B). Flow cytometry result showed that CYCLIN E1 expression increased the percentage of S1 phase cell released after double-thymidine block, while SPOP expression reduced the percentage and rescued the CYCLIN E1 phenotype (Sup. Fig. S3C). In the normal cell cycle assay without synchronization, wild-type SPOP, but not SPOP mutants, reversed the S phase increase by CYCLIN E1 (Sup. Fig. S3D). Cell proliferation assay studied with RTCA indicated that CYCLIN E1 increased cell proliferation rate and SPOP expression inhibited it (Fig. 5d). Cell migration assay with RTCA also showed that CYCLIN E1 accelerated cell migration while SPOP expression rescued the phenotype (Fig. 5e). The colony formation assay showed that CYCLIN E1 increased the colony number and SPOP expression rescued it (Fig. 5f). To study their ability in tumor formation, the above four cell lines were injected into nude mice. The results showed that CYCLIN E1 increased tumor volume and SPOP expression greatly repressed it; importantly, SPOP expression reversed the effect caused by CYCLIN E1 overexpression (Fig. 5g, left). Western blotting confirmed that the tumors were developed from the original cell lines (Fig. 5g, right). These data nicely fit the previously published data about CYCLIN E1’s roles in tumor, and indicated that SPOP acts as a tumor-suppressor through inhibiting CYCLIN E1 functions.

### SPOP increases the tumor number formed by CCRC cells

**Fig. 6:**
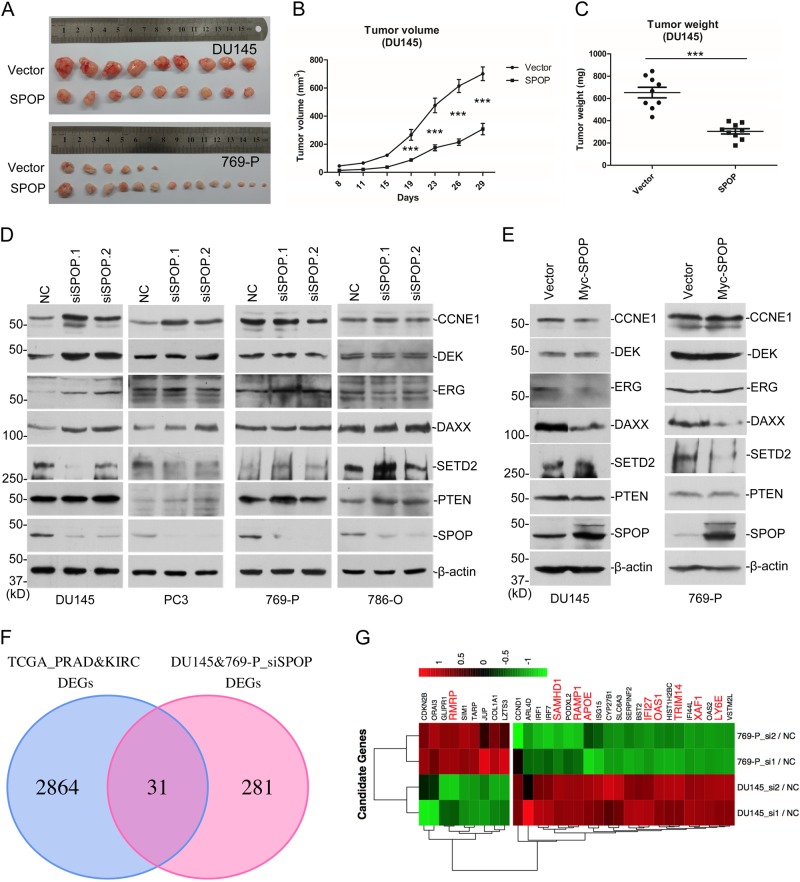
SPOP plays opposite roles in PCa and CCRC tumorigenesis. **a–c** Xenograft experiments were performed in nude mice with DU145 or 769-P cells stable expressing SPOP. The resulted tumor tissues were pictured (**a**). Tumor volumes (**b**) and weights (**c**) of DU145 were shown as mean ± SEM, *n* = 9. ***means *p* < 0.005. **d** DU145, PC3, 769-P, and 786-O cells were transfected with control or two independent *SPOP*-specific siRNAs. Seventy-two hours after transfection, cells were harvested for WB. **e** DU145 or 769-P cells infected with lentiviral empty vector or SPOP was assayed with WB. **f** Venn-diagram shows the overlapped genes of DEGs between TCGA human cancer samples (PCa+CRCC) and DEGs of SPOP RNAi (DU145+769-P). **g** Heat map of the overlapped genes in **f**. Cancer-related genes were labeled with red color

Previously we have revealed that SPOP promotes the degradation of SETD2, an important tumor-suppressor in many cancers, through poly-ubiquitination [12]. Other groups also reported SPOP to be an oncogene in CCRC [9, 23]. Then a debate about SPOP’s role in tumorigenesis exists in the field. We have discovered two substrates for SPOP, one oncogene and one tumor-suppressor, which led us to investigate whether SPOP play opposite roles in PCa and CCRC. We stably expressed SPOP in 769-P, a widely used CCRC cell line, and performed xenograft experiments in nude mice, side by side with SPOP stable DU145 cells. The results suggest SPOP expression repressed tumor volume and weight of DU145, same as previous observation (Figs. 5g and 6a–c), and increased the tumor number formed by 769-P (Fig. 6a). Interestingly, SPOP expression did not affect the average tumor size or weight of 769-P (data not shown), suggesting SPOP may be only involved in the early stage of kidney tumor formation. Western blotting was carried out to confirm the tumors were developed from original 769-P stable cells (Sup. Fig. S4A). These data agree with previous publications and provided strong evidence that SPOP plays opposite roles in tumor formation from PCa and CCRC cell lines.

### SPOP selectively regulates substrates stability in PCa and CCRC cell lines

We then speculated that SPOP may exert its functions through selectively regulates the substrates’ stability in different cell lines. We knocked down *SPOP* with siRNAs in two PCa cell lines, DU145 and PC3, and two CCRC cell lines, 786-O and 769-P. In PCa cell lines, SPOP deficiency caused the increase of CYCLIN E1, DEK, ERG, DAXX, but not SETD2 and PTEN (Fig. 6d). In CCRC cell lines, it was absolutely opposite, and only SETD2 and PTEN were stabilized but not the others (Fig. 6d). When SPOP was overexpressed in DU145, CYCLIN E1, ERG and DAXX were decreased; while in 769-P, SPOP expression decreased the level of SETD2 and DAXX (Fig. 6e). Both the RNAi and overexpression experiments indicated that SPOP selectively regulates substrates protein levels in PCa and CCRC cell lines.

### Distinguished gene expression profiles regulated by SPOP in PCa and CCRC cell lines

To further elucidate the different roles of SPOP in two cancers, we studied the global gene expression patterns with RNA-Seq. Only a small portion of differential expressed genes (DEGs, ≥ 2-folds) after *SPOP* knockdown in DU145 and 769-P were overlapped. We then analyzed DEGs ≥ 1.5-folds, and identified only 37 genes increased in both cell lines (Sup. Fig. S4B, C), suggesting the gene expression profiles regulated by SPOP in two cell lines are largely different. The gene ontology studies about the DEGs suggest SPOP regulates different signaling programs in DU145 and 769-P cell lines (Sup. Fig. S4D–G). We analyzed the gene expression data of PCa and CCRC in TCGA, and identified totally 2895 DEGs between cancer and adjacent tissues in two types of cancers. We overlapped these DEGs with all those regulated by SPOP in the above two cell lines, and identified 31 genes (Fig. 6f). These 31 genes may be related with tumorigenesis and development process regulated by SPOP. A heat map of their fold changes after *SPOP* knockdown showed that they are oppositely regulated by SPOP in DU145 and 769-P cell lines (Fig. 6g), which further suggests SPOP has different functions in two cancers. We searched the literature and identified nine known cancer-related genes among the 31 genes, including lncRNA-RMRP, SAMHD1, RAMP1, APOE, IFI27, OAS1, TRIM14, XAF1, and LY6E (Fig. 6g). The mRNA levels of the nine genes were further verified with quantitative RT-PCR (Sup. Fig. S4H). These genes may be involved in the selective regulation by SPOP of tumorigenesis in PCa and CCRC.

## Discussion

SPOP is one of the most frequent mutated genes in PCa. In the current study, we identified CYCLIN E1 as its substrate. SPOP directly interacts with CYCLIN E1, poly-ubiquitinates CYCLIN E1 in vivo and in vitro, and represses cancer-related phenotypes induced by CYCLIN E1 expression. Thus, we reveal a new mechanism for SPOP in repressing PCa tumorigenesis.

An early structural study revealed a consensus sequence in the substrates for SPOP recognition [3]. In our study, we also mapped the whole CYCLIN E1 sequence for SPOP-binding sites. We constructed a series of point and deletion mutants and found that the final constructs, which completely abolished the ability of binding SPOP, almost mutated around half of the amino acids in the cyclin domain. So we thought that probably the entire cyclin domain is critical for the binding. A recent study also showed a novel SPOP recognition motif different from the identified consensus sequence [32]. These data together suggest that a new rule probably exists for SPOP-substrate binding.

CYCLIN E1 is one of the important proteins for cell cycle progression and it is well-known that CYCLIN E1 couples with CDK2 and regulates G1/S transition and DNA replication [33, 34]. Moreover, CDK2-indepenent function for CYCLIN E1 has been reported by several groups, which is critical for proper MCM complex loading to replication origins [35–37]. Then it is also important to regulate CDK2-free CYCLIN E1 to prevent from abnormal DNA replication. FBXW7 regulates CYCLIN E1 stability, which is phosphorylated by CDK2 [26, 29]. RHOBTB3 is a Golgi-associated protein, which targets CYCLIN E1 and regulates the S/G2 transition [27]. The ubiquitin E3 ligase for CDK2-free CYCLIN E1 in nuclear is unknown. We showed here that SPOP regulates CYCLIN E1 protein level in the nuclear; CDK2 competes with SPOP for CYCLIN E1 binding, which inhibits CYCLIN E1 poly-ubiquitination and degradation by SPOP; the SPOP-interacting domain in CYCLIN E1 was mapped to cyclin domain, which is responsible for CDK2 interaction; and SPOP promoted the degradation of CYCLIN E1 with phosphorylation site mutation. All these data indicate that SPOP/CUL3/RBX1 complex poly-ubiquitinates CDK2-free CYCLIN E1 in nuclear, which is important for proper cell cycle progression. Inappropriate degradation of CYCLIN E1 on chromatin may lead to abnormal DNA replication and increase gene mutations in the cell, which will increase the risk of tumorigenesis [38].

Our data showed that SPOP regulates CYCLIN E1 stability specific in some prostate and bladder cancer cell lines, but not in the other tested cell lines. SPOP overexpression could successfully decrease CYCLIN E1 in nearly all the tested cells. In some cell lines, CYCLIN E1 even decreased after SPOP knockdown, suggesting some unknown factors involves in regulating CYCLIN E1 stability with SPOP in a cell type dependent manner. We have further showed that SPOP selectively regulates its substrates in PCa and CCRC cell lines. Importantly, the xenograft experiments showed that SPOP represses tumor growth rising from a PCa cell line and enhances tumor formation of a CCRC cell line. A recent study indicated that SPOP mutants has cancer specific preference in endometrial cancer to degrade BET family proteins [16]. Our data suggest that wild-type SPOP acts oppositely in PCa and CCRC, probably through selective substrate degradation. Thus, therapeutic efforts to control SPOP activity in CCRC will perhaps increase the possibility of PCa. So it is important to further elucidate the underlying mechanism that determines SPOP selectivity, and carefully evaluate the effects of SPOP regulators in both cancers. Meanwhile, it will be necessary to determine SPOP’s real function in other cancers during the future studies, and our data will be helpful to clarify it. Our gene expression analysis revealed that SPOP regulates different biological processes and signaling pathways in 769-P and DU145 and identified some candidate genes, including lncRNA-RMRP, SAMHD1, RAMP1, APOE, IFI27, OAS1, TRIM14, XAF1, and LY6E. The above protein and mRNA analysis may provide important clues for future mechanistic studies of SPOP in PCa and CCRC, as well as to help to characterize SPOP’s function in other cells or cancers. SPOP usually exists in the nuclear and targets many proteins on chromatin, including SETD2, BRD4, DAXX and so on, which could contribute to the selective transcription in different cell lines.

## Methods

### Cell lines and reagents

HEK293, HeLa, HCT116, HepG2, DU145, UMUC3, and HL7702 cell lines were cultured in Dulbecco's Modified Eagle Medium (DMEM) supplemented with 10% fetal bovine serum (FBS), 1% penicillin and streptomycin. ACHN, 786-O, 769-P, PC3, SV-HUC-1, T24, U2OS, and 22RV1 cell lines were cultured in RPMI 1640 supplemented with 10% FBS, 1% penicillin and streptomycin. Caki-1 cell lines were cultured in McCOY’s 5A supplemented with 10% FBS, 1% penicillin and streptomycin. All the cell lines were purchased from Cell Bank of Chinese Academy of Science. The tests for mycoplasma contamination were performed by the vendor.

Antibodies against Flag (Sigma F3165), HA (Origene TA100012), Myc (Abclonal AE038), EGFP (Abmart 264076), β-Actin (Abclonal AC004), CUL3 (Epitomics #2489-1), RBX1 (Epitomics #5296-1), CYCLIN E1 (CST #4129, Epitomics #3327-1, Bioworld BS1085, Abcam ab3927), ERG (Abclonal A1240), DEK (BD 610948), DAXX (CST #4533), PTEN (Abclonal A2113), FBXW7 (Abclonal A5872), RHOBTB3 (LSBIO LS-C385850) were purchased from indicated commercial sources. Rabbit anti-SETD2 was raised and now commercial available at Abclonal. Mouse anti-SPOP was raised at the Wuhan Institute of Virology, CAS.

### Real-time cell analysis (RTCA) of cell proliferation and migration

Cell proliferation and migration were analyzed with RTCA assay as described before [39]. Cells were cultured at 6000 per well in CIM-Plate wells coated with (invasion) or without (migration) matrigel. The cell index signals were read by xCELLigence RTCA DP Analyzer (ACEA bioscience Inc). Invasion and migration are monitored continuously over a 48-hour period. Each experiment was repeated three times and results were presented as mean ± SD.

### Pipeline of RNA-seq analysis

Two biological replicates were sequenced for each sample. mRNA-seq library was performed by using Illumina Tru-Seq library construction kit. A 5 μg of total RNA was used as initiation and then prepared according to the manufacturer’s instruction. mRNA-seq libraries were sequenced using HiSeq2000 for 100-bp paired-end sequencing. Quality control of mRNA-seq data was performed using Fatsqc, and then low quality bases were trimmed. After quality control, data were mapped to hg19 genome reference by Tophat2 and allow maximum 2 mismatch. Cufflinks were used to find out differential expression genes. Gene ontology analysis was performed using DAVID (http://david.abcc.ncifcrf.gov).

### Survival analysis

The disease free survival (DFS, also called relapse-free survival and RFS) analysis based on gene expression via GEPIA performs (http://gepia.cancer-pku.cn/) [40]. GEPIA uses log-rank test, also called the Mantel–Cox test, for the hypothesis evaluation. The cox proportional hazard ratio is based on Cox PH Model. The datasets of prostate cancer are based on TCGA-PRAD (Prostate adenocarcinoma).

### Data access

The original RNA-Seq data were submitted to the GEO database at the following link, https://www.ncbi.nlm.nih.gov/geo/query/acc.cgi?acc=GSE100743.

## Electronic supplementary material

Supplemental Materials

## References

[1] Nagai Y, Kojima T, Muro Y, Hachiya T, Nishizawa Y, Wakabayashi T (1997). Identification of a novel nuclear speckle-type protein, SPOP. FEBS Lett.

[2] Marzahn MR, Marada S, Lee J, Nourse A, Kenrick S, Zhao H (2016). Higher-order oligomerization promotes localization of SPOP to liquid nuclear speckles. EMBO J.

[3] Zhuang M, Calabrese MF, Liu J, Waddell MB, Nourse A, Hammel M (2009). Structures of SPOP-substrate complexes: insights into molecular architectures of BTB-Cul3 ubiquitin ligases. Mol Cell.

[4] Zhang Q, Zhang L, Wang B, Ou CY, Chien CT, Jiang J (2006). A hedgehog-induced BTB protein modulates hedgehog signaling by degrading Ci/Gli transcription factor. Dev Cell.

[5] Kwon JE, La M, Oh KH, Oh YM, Kim GR, Seol JH (2006). BTB domain-containing speckle-type POZ protein (SPOP) serves as an adaptor of Daxx for ubiquitination by Cul3-based ubiquitin ligase. J Biol Chem.

[6] Hernandez-Munoz I, Lund AH, van der Stoop P, Boutsma E, Muijrers I, Verhoeven E (2005). Stable X chromosome inactivation involves the PRC1 Polycomb complex and requires histone MACROH2A1 and the CULLIN3/SPOP ubiquitin E3 ligase. Proc Natl Acad Sci USA.

[7] Geng C, He B, Xu L, Barbieri CE, Eedunuri VK, Chew SA (2013). Prostate cancer-associated mutations in speckle-type POZ protein (SPOP) regulate steroid receptor coactivator 3 protein turnover. Proc Natl Acad Sci USA.

[8] Li C, Ao J, Fu J, Lee DF, Xu J, Lonard D (2011). Tumor-suppressor role for the SPOP ubiquitin ligase in signal-dependent proteolysis of the oncogenic co-activator SRC-3/AIB1. Oncogene.

[9] Li G, Ci W, Karmakar S, Chen K, Dhar R, Fan Z (2014). SPOP promotes tumorigenesis by acting as a key regulatory hub in kidney cancer. Cancer Cell.

[10] Gan W, Dai X, Lunardi A, Li Z, Inuzuka H, Liu P (2015). SPOP Promotes Ubiquitination and degradation of the ERG oncoprotein to suppress prostate cancer progression. Mol Cell.

[11] Theurillat JP, Udeshi ND, Errington WJ, Svinkina T, Baca SC, Pop M (2014). Prostate cancer. Ubiquitylome analysis identifies dysregulation of effector substrates in SPOP-mutant prostate cancer. Science.

[12] Zhu K, Lei PJ, Ju LG, Wang X, Huang K, Yang B (2017). SPOP-containing complex regulates SETD2 stability and H3K36me3-coupled alternative splicing. Nucl Acids Res.

[13] Wu F, Dai X, Gan W, Wan L, Li M, Mitsiades N (2017). Prostate cancer-associated mutation in SPOP impairs its ability to target Cdc20 for poly-ubiquitination and degradation. Cancer Lett.

[14] Jin X, Wang J, Gao K, Zhang P, Yao L, Tang Y (2017). Dysregulation of INF2-mediated mitochondrial fission in SPOP-mutated prostate cancer. PLoS Genet.

[15] Zhang P, Wang D, Zhao Y, Ren S, Gao K, Ye Z (2017). Intrinsic BET inhibitor resistance in SPOP-mutated prostate cancer is mediated by BET protein stabilization and AKT-mTORC1 activation. Nat Med.

[16] Janouskova H, El Tekle G, Bellini E, Udeshi ND, Rinaldi A, Ulbricht A (2017). Opposing effects of cancer-type-specific SPOP mutants on BET protein degradation and sensitivity to BET inhibitors. Nat Med.

[17] Dai X, Wang Z, Wei W (2017). SPOP-mediated degradation of BRD4 dictates cellular sensitivity to BET inhibitors. Cell Cycle.

[18] Dai X, Gan W, Li X, Wang S, Zhang W, Huang L (2017). Prostate cancer-associated SPOP mutations confer resistance to BET inhibitors through stabilization of BRD4. Nat Med.

[19] Geng C, Rajapakshe K, Shah SS, Shou J, Eedunuri VK, Foley C (2014). Androgen receptor is the key transcriptional mediator of the tumor suppressor SPOP in prostate cancer. Cancer Res.

[20] Barbieri CE, Baca SC, Lawrence MS, Demichelis F, Blattner M, Theurillat JP (2012). Exome sequencing identifies recurrent SPOP, FOXA1 and MED12 mutations in prostate cancer. Nat Genet.

[21] Mani RS (2014). The emerging role of speckle-type POZ protein (SPOP) in cancer development. Drug Discov Today.

[22] Stone L (2016). Kidney cancer: On target-inhibiting SPOP in ccRCC. Nat Rev Urol.

[23] Liu J, Ghanim M, Xue L, Brown CD, Iossifov I, Angeletti C (2009). Analysis of Drosophila segmentation network identifies a JNK pathway factor overexpressed in kidney cancer. Science.

[24] Lawrence MS, Stojanov P, Mermel CH, Robinson JT, Garraway LA, Golub TR (2014). Discovery and saturation analysis of cancer genes across 21 tumour types. Nature.

[25] Guo ZQ, Zheng T, Chen B, Luo C, Ouyang S, Gong S (2016). Small-molecule targeting of E3 ligase adaptor SPOP in kidney cancer. Cancer Cell.

[26] Koepp DM, Schaefer LK, Ye X, Keyomarsi K, Chu C, Harper JW (2001). Phosphorylation-dependent ubiquitination of cyclin E by the SCFFbw7 ubiquitin ligase. Science.

[27] Lu A, Pfeffer SR (2013). Golgi-associated RhoBTB3 targets cyclin E for ubiquitylation and promotes cell cycle progression. J Cell Biol.

[28] Strohmaier H, Spruck CH, Kaiser P, Won KA, Sangfelt O, Reed SI (2001). Human F-box protein hCdc4 targets cyclin E for proteolysis and is mutated in a breast cancer cell line. Nature.

[29] Bhaskaran N, van Drogen F, Ng HF, Kumar R, Ekholm-Reed S, Peter M (2013). Fbw7alpha and Fbw7gamma collaborate to shuttle cyclin E1 into the nucleolus for multiubiquitylation. Mol Cell Biol.

[30] Welcker M, Singer J, Loeb KR, Grim J, Bloecher A, Gurien-West M (2003). Multisite phosphorylation by Cdk2 and GSK3 controls cyclin E degradation. Mol Cell.

[31] Clurman BE, Sheaff RJ, Thress K, Groudine M, Roberts JM (1996). Turnover of cyclin E by the ubiquitin-proteasome pathway is regulated by cdk2 binding and cyclin phosphorylation. Genes Dev.

[32] Zhang J, Bu X, Wang H, Zhu Y, Geng Y, Nihira NT (2018). Cyclin D-CDK4 kinase destabilizes PD-L1 via cullin 3-SPOP to control cancer immune surveillance. Nature.

[33] Spoerri L, Gabrielli B (2013). Similar, not the same: diverse roles and regulation of cyclin Es. Cell Cycle.

[34] Siu KT, Rosner MR, Minella AC (2012). An integrated view of cyclin E function and regulation. Cell Cycle.

[35] Geng Y, Lee YM, Welcker M, Swanger J, Zagozdzon A, Winer JD (2007). Kinase-independent function of cyclin E. Mol Cell.

[36] Mazumder S, Plesca D, Almasan A (2007). A jekyll and hyde role of cyclin E in the genotoxic stress response: switching from cell cycle control to apoptosis regulation. Cell Cycle.

[37] Hu W, Nevzorova YA, Haas U, Moro N, Sicinski P, Geng Y (2014). Concurrent deletion of cyclin E1 and cyclin-dependent kinase 2 in hepatocytes inhibits DNA replication and liver regeneration in mice. Hepatology.

[38] Teixeira LK, Wang X, Li Y, Ekholm-Reed S, Wu X, Wang P (2015). Cyclin E deregulation promotes loss of specific genomic regions. Curr Biol.

[39] Zhao QY, Lei PJ, Zhang X, Zheng JY, Wang HY, Zhao J (2016). Global histone modification profiling reveals the epigenomic dynamics during malignant transformation in a four-stage breast cancer model. Clin Epigenetics.

[40] Tang Zefang, Li Chenwei, Kang Boxi, Gao Ge, Li Cheng, Zhang Zemin (2017). GEPIA: a web server for cancer and normal gene expression profiling and interactive analyses. Nucleic Acids Research.

