# Supplementary material for: SPOP suppresses prostate cancer through regulation of CYCLIN E1 stability
Source: Cell Death Differ. 2018 Sep 20;26(6):1156–68. doi: 10.1038/s41418-018-0198-0 (PMC6748103; doi:10.1038/s41418-018-0198-0)
Supplement: Supplementary file 1 — Supplemental Materials [file 41418_2018_198_MOESM1_ESM.pdf]

Sup. Figure 1

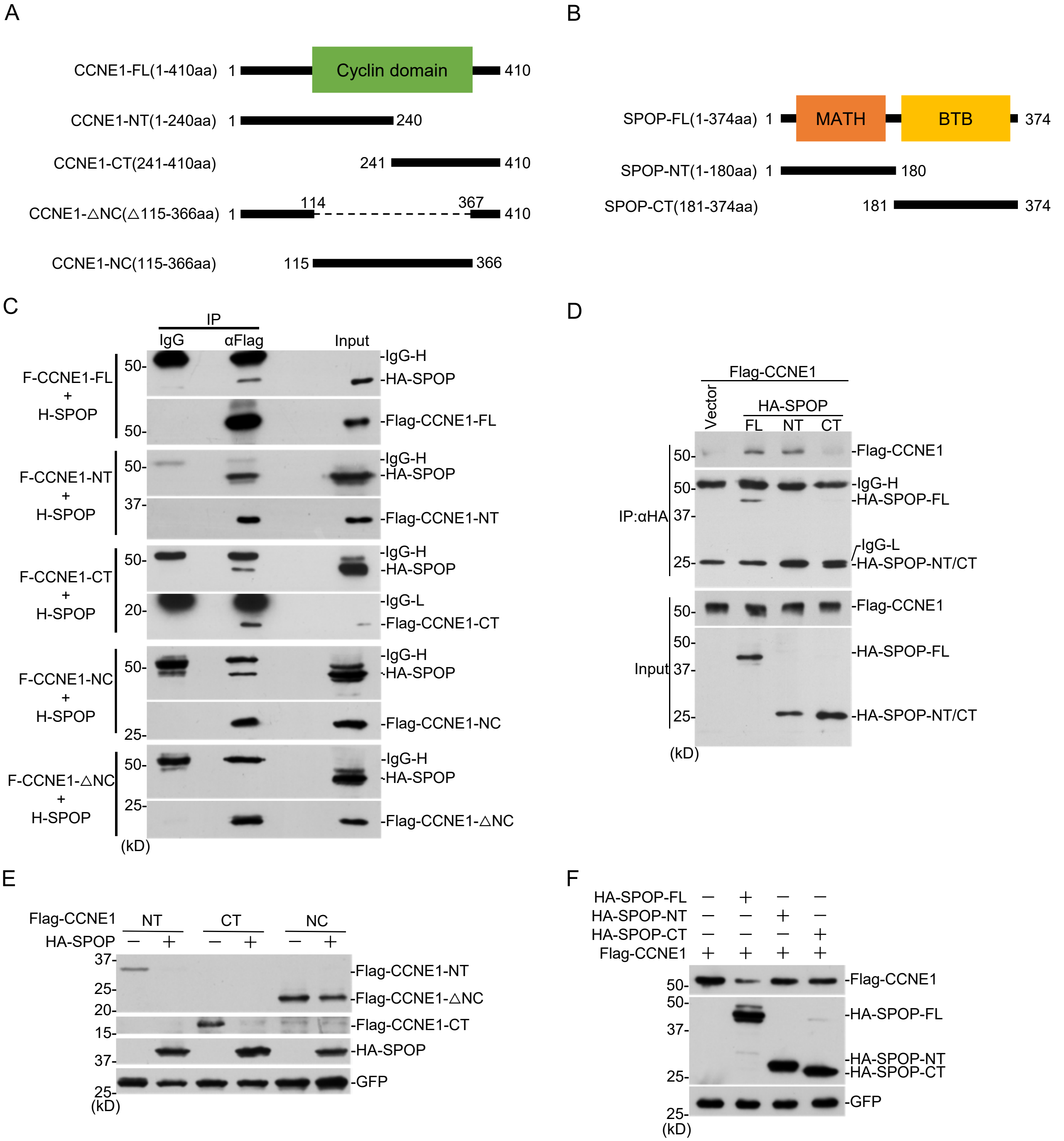
A
CCNE1-FL(1-410aa) 1
Cyclin domain
410
CCNE1-NT(1-240aa) 1
240
CCNE1-CT(241-410aa)
241
410
CCNE1-△NC(△115-366aa) 1
114
367
410
CCNE1-NC(115-366aa)
115
366
B
SPOP-FL(1-374aa) 1
MATH
BTB
374
SPOP-NT(1-180aa) 1
180
SPOP-CT(181-374aa)
181
374
C
IP
IgG
αFlag
Input
F-CCNE1-FL
+
H-SPOP
F-CCNE1-NT
+
H-SPOP
F-CCNE1-CT
+
H-SPOP
F-CCNE1-NC
+
H-SPOP
F-CCNE1-△NC
+
H-SPOP
-IgG-H
-HA-SPOP
-Flag-CCNE1-FL
-Flag-CCNE1-NT
-IgG-L
-Flag-CCNE1-CT
-Flag-CCNE1-NC
-Flag-CCNE1-△NC
(kD)
D
Flag-CCNE1
Vector
HA-SPOP
FL
NT
CT
IP:αHA
Input
-Flag-CCNE1
-IgG-H
-HA-SPOP-FL
-IgG-L
-HA-SPOP-NT/CT
(kD)
E
Flag-CCNE1
NT
CT
NC
HA-SPOP
-Flag-CCNE1-NT
-Flag-CCNE1-△NC
-Flag-CCNE1-CT
-HA-SPOP
-GFP
(kD)
F
HA-SPOP-FL
HA-SPOP-NT
HA-SPOP-CT
Flag-CCNE1
-Flag-CCNE1
-HA-SPOP-FL
-HA-SPOP-NT
-HA-SPOP-CT
-GFP
(kD)

Sup. Figure 2

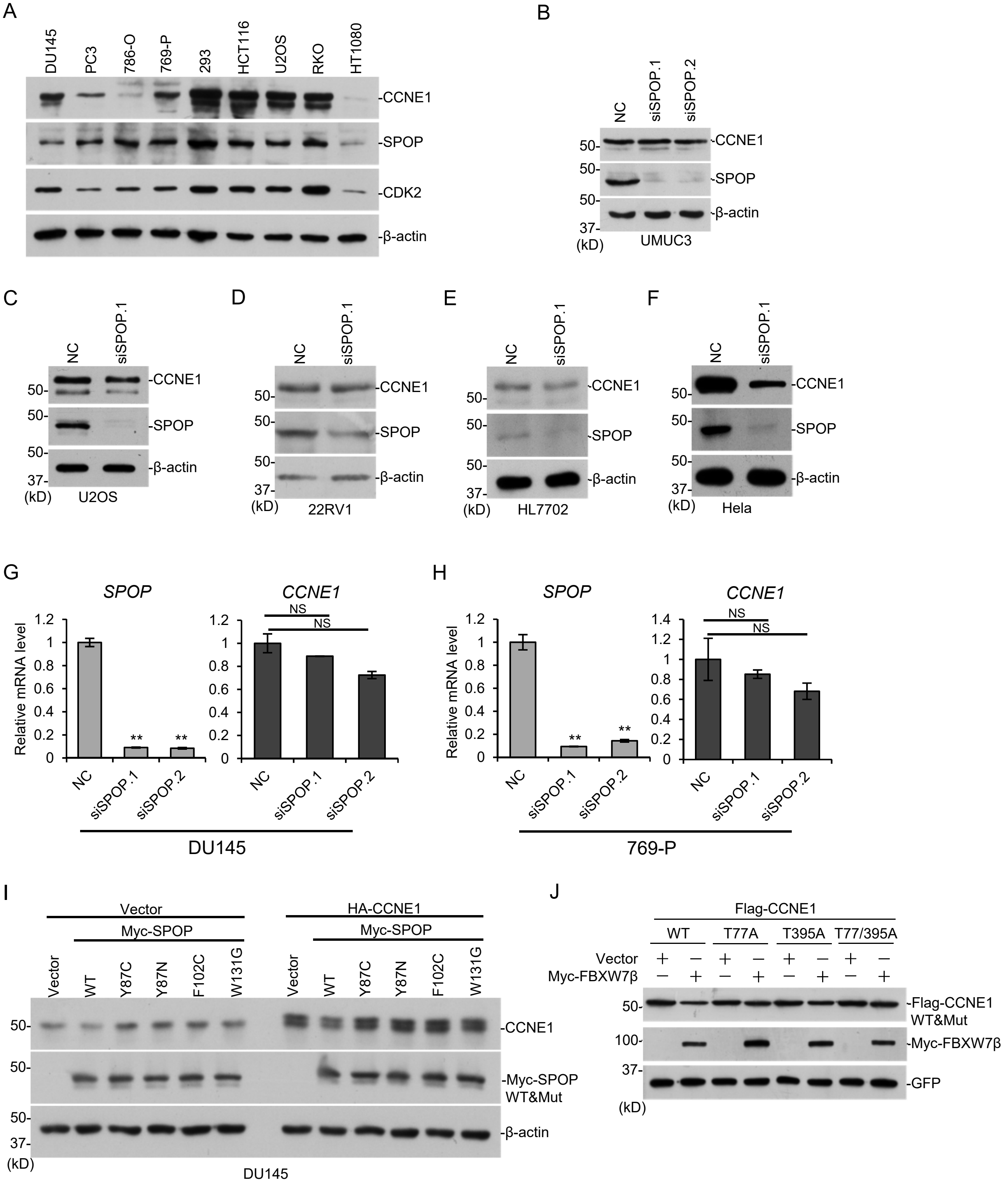
A
DU145
PC3
786-O
769-P
293
HCT116
U2OS
RKO
HT1080
-CCNE1
-SPOP
-CDK2
-β-actin
B
NC
siSPOP.1
siSPOP.2
-CCNE1
-SPOP
-β-actin
(kD)
UMUC3
C
U2OS
D
22RV1
E
HL7702
F
Hela
G
SPOP
CCNE1
Relative mRNA level
NS
**
DU145
H
769-P
I
Vector
HA-CCNE1
Myc-SPOP
WT
Y87C
Y87N
F102C
W131G
-Myc-SPOP
WT&Mut
DU145
J
Flag-CCNE1
T77A
T395A
T77/395A
Myc-FBXW7β
-Flag-CCNE1
WT&Mut
-Myc-FBXW7β
-GFP

Sup. Figure 3

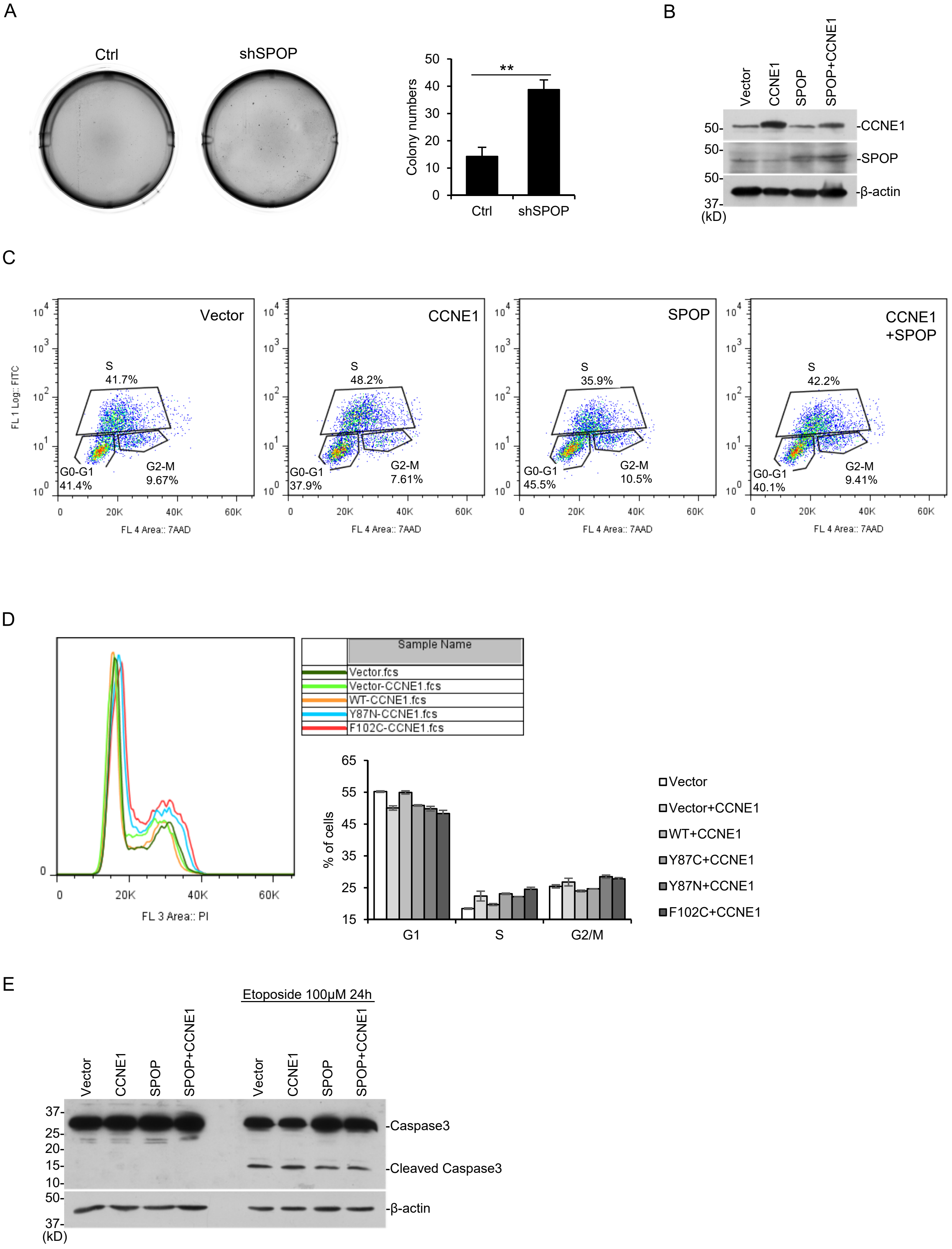
A
Ctrl
shSPOP
Colony numbers
**
50
40
30
20
10
0
Ctrl
shSPOP
B
Vector
CCNE1
SPOP
SPOP+CCNE1
50-
-CCNE1
50-
-SPOP
50-
-β-actin
37-
(kD)
C
FL 1 Log:: FITC
Vector
S
41.7%
G0-G1
41.4%
G2-M
9.67%
CCNE1
S
48.2%
G0-G1
37.9%
G2-M
7.61%
SPOP
S
35.9%
G0-G1
45.5%
G2-M
10.5%
CCNE1
+SPOP
S
42.2%
G0-G1
40.1%
G2-M
9.41%
0
20K
40K
60K
FL 4 Area:: 7AAD
D
Sample Name
Vector.fcs
Vector-CCNE1.fcs
WT-CCNE1.fcs
Y87N-CCNE1.fcs
F102C-CCNE1.fcs
0
20K
40K
60K
FL 3 Area:: PI
% of cells
65
55
45
35
25
15
G1
S
G2/M
Vector
Vector+CCNE1
WT+CCNE1
Y87C+CCNE1
Y87N+CCNE1
F102C+CCNE1
E
Etoposide 100μM 24h
Vector
CCNE1
SPOP
SPOP+CCNE1
Vector
CCNE1
SPOP
SPOP+CCNE1
37-
25-
20-
15-
10-
-Caspase3
-Cleaved Caspase3
50-
37-
-β-actin
(kD)

Sup. Figure 4

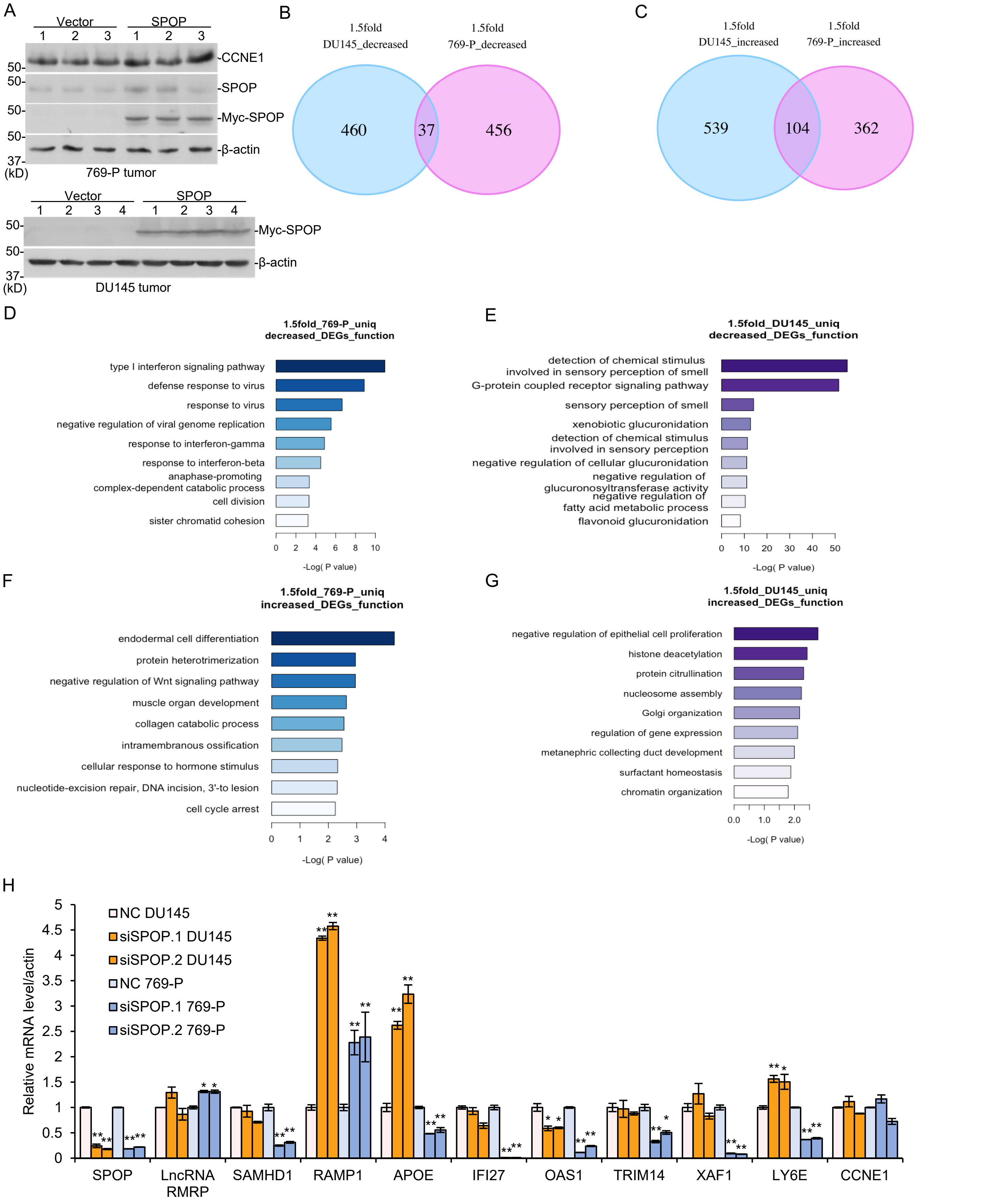
A
Vector
SPOP
1 2 3 1 2 3
-CCNE1
-SPOP
-Myc-SPOP
-β-actin
50-
50-
50-
50-
37-
(kD)
769-P tumor
Vector
SPOP
1 2 3 4 1 2 3 4
-Myc-SPOP
-β-actin
(kD)
DU145 tumor
B
1.5fold DU145_decreased
1.5fold 769-P_decreased
460
37
456
C
1.5fold DU145_increased
1.5fold 769-P_increased
539
104
362
D
1.5fold_769-P_uniq decreased_DEGs_function
type I interferon signaling pathway
defense response to virus
response to virus
negative regulation of viral genome replication
response to interferon-gamma
response to interferon-beta
anaphase-promoting complex-dependent catabolic process
cell division
sister chromatid cohesion
-Log(P value)
E
1.5fold_DU145_uniq decreased_DEGs_function
detection of chemical stimulus involved in sensory perception of smell
G-protein coupled receptor signaling pathway
sensory perception of smell
xenobiotic glucuronidation
detection of chemical stimulus involved in sensory perception
negative regulation of cellular glucuronidation
negative regulation of glucuronosyltransferase activity
negative regulation of fatty acid metabolic process
flavonoid glucuronidation
-Log(P value)
F
1.5fold_769-P_uniq increased_DEGs_function
endodermal cell differentiation
protein heterotrimerization
negative regulation of Wnt signaling pathway
muscle organ development
collagen catabolic process
intramembranous ossification
cellular response to hormone stimulus
nucleotide-excision repair, DNA incision, 3'-to lesion
cell cycle arrest
-Log(P value)
G
1.5fold_DU145_uniq increased_DEGs_function
negative regulation of epithelial cell proliferation
histone deacetylation
protein citrullination
nucleosome assembly
Golgi organization
regulation of gene expression
metanephric collecting duct development
surfactant homeostasis
chromatin organization
-Log(P value)
H
Relative mRNA level/actin
NC DU145
siSPOP.1 DU145
siSPOP.2 DU145
NC 769-P
siSPOP.1 769-P
siSPOP.2 769-P
SPOP
LncRNA RMRP
SAMHD1
RAMP1
APOE
IFI27
OAS1
TRIM14
XAF1
LY6E
CCNE1

Supplemental figure legends

Fig. S1 The interacting domains between SPOP and CYCLIN E1. A & B. Schematic maps of domains and constructed truncations for CYCLIN E1 (A) and SPOP (B). **C.** HEK293 cells were transfected with indicated plasmids followed by treatment with 10 μM MG132 for 12 hr. Co-IP was performed to map the CYCLIN E1 domains interacting with SPOP. **D.** HEK293 cells were transfected with indicated plasmids for followed by treatment with 10 μM MG132 for 12 hr. Co-IP was performed to study SPOP domains interacting with CYCLIN E1. **E & F.** HEK293 cells were transfected with indicated plasmids. After 24 hr, cells were harvested and CYCLIN E1 protein level was assayed with WB.

Fig. S2 SPOP regulates endogenous CYCLIN E1 in specific cell lines. A. Protein levels of CYCLIN E1, SPOP and CDK2 in the indicated cell lines. **B-F.** UMUC3, U2OS, 22RV1, HL7702, Hela cells were transfected with control or SPOP-specific siRNAs. After 72 hr, cells were harvested for WB. **G & H.** Real-time PCR analysis to examine *CCNE1* and *SPOP* mRNA levels after depletion of SPOP in DU145 (G) and 769-P (H). NS = Not significant. **I.** Wild type and mutated SPOP were exogenous expressed in DU145, the level of CYCLIN E1 was measured with western blotting. **J.** CYCLIN E1 wild type and mutants T77A, T395A, T77/395A were transfected into DU145 with or without FBXW7β, and western blotting was carried out with the indicated antibodies. GFP was co-transfected and blotted as control for transfection efficiency.

Fig. S3 SPOP regulates CYCLIN E1-mediated cell cycle control. A. soft agar colony formation assay was performed with SPOP stable knockdown cells. Colony number was counted and plotted as indicated. ** means p value < 0.01. **B.** *SPOP* and *CCNE1* stable expressed DU145 cell lines were generated and validated with western blotting. **C.** Cell lines in (B) were synchronized with double-thymidine block and released for 2 hr. BrdU-incorporation assay were performed and analyzed with flow cytometry. **D.** *CCNE1* was co-expressed with SPOP wild type or mutants in DU145 cells and cell cycle was analyzed with flow cytometry. **E.** The indicated stable cell lines were treated

with or without etoposide, antibodies recognizing caspase 3 were used to detect the procaspase 3 and cleaved caspase 3.

Fig. S4 The bioinformatics analysis of SPOP-induced change of gene expression. A. Western blotting of three random-chosen tumors derived from 769-P cells, and four random tumors from DU145 cells. **B & C.** Venn diagram shows the overlapped gene number of decreased (B) or increased (C) DEGs in DU145 and 769-P cells after SPOP knockdown. **D.** GO analysis of the decreased DEGs unique in 769-P cells. **E.** GO analysis of the decreased DEGs unique in DU145 cells. **F.** GO analysis of the increased DEGs unique in 769-P cells. **G.** GO analysis of the increased DEGs unique in DU145 cells. **H.** Real-time PCR analysis to verify the expression of the identified genes in Fig.7F.

Supplemental Methods

Protein expression in bacteria and GST purification

Individual cDNA sequences were cloned into pGEX-KG vector. The constructs were transformed into BL-21 bacteria, which were induced with 0.2 mM IPTG at 18°C for 4 hr. The harvested cells were sonicated and the lysates were centrifuged at 10,000×g for 1h. Recombinant proteins were purified from the supernatant with Glutathione Sepharose 4 according to manufacturer's instructions (GE Healthcare). Protein concentration was quantified by Qubit 2.0 (Invitrogen).

Immunoprecipitation

Cells were harvested and lysed in NP40 Lysis buffer (50 mM Tris, pH 7.4, 150 mM NaCl, 0.5% NP40) or high salt lysis buffer (20 mM HEPES pH 7.4, 10% glycerol, 0.35 M NaCl, 1 mM MgCl2, 0.5% triton X-100, 1 mM DTT) in the presence of proteinase inhibitors. After removing insoluble particles, the supernatant was incubated with protein G beads (GE Healthcare) and specific antibody at 4°C for 4 hr. The beads were spin down and washed three times with lysis buffer. After the final wash, SDS loading buffer was added to the beads to release proteins for SDS-PAGE and Western blotting.

Immunofluorescent staining

Cells were cultured on cover slips and fixed with freezing methanol after wash twice in PBS. The cover slips were then washed three times by PBS and blocked in PBS with 1% BSA for 10 min or one hour. The cover slips then were hybridized with first and second antibodies for one hour, respectively. Then the slips were mounted with prolong anti-fade kit (Invitrogen) and observed with fluorescent microscopy.

GST pulldown assays

GST-CCNE1 or GST was incubated with glutathione–sepharose beads in binding buffer (50 mM HEPES (pH 7.4), 150 mM KCl, 2.5 mM MgCl2, 5% Glycerol,

1% BSA (bovine serum albumin) at 4°C for 1 hr. The beads were then washed twice with binding buffer, and mixed with His-tagged proteins. Samples were incubated at 4°C for 1 h and followed by washing 3 times with binding buffer. Finally, SDS loading buffer was added and samples were heated to 95◦C for 5 min for SDS-PAGE and Western blot analysis.

Reverse transcription and quantitative PCR

The indicated cells were harvested and the total RNA was extracted with RNA extraction kit (Aidlab) according to the manufacturer's manual. The amount of mRNA was quantified with Qubit (Invitrogen). Approximately 1ug of total RNA was used for reverse transcription with a first strand cDNA synthesis kit (Toyobo). Real-time PCR was then performed with My-IQ (Biorad) according to the manufacturer's standard protocol. β-actin was used to normalize the amount of each sample. Assays were repeated at least three times (n = 3). Data shown were average values ± SD. Primer sequences are presented in Supplemental Table S1.

Protein expression by baculovirus in insect cells

Proteins was expressed and purified by using Invitrogen Bac-to-Bac® baculovirus expression system. The cDNA sequences of CUL3, RBX1 and SPOP were cloned into pFastBac vector. The plasmids were transfected into DH10BacTM cells, then plates were incubated for 48 hours at 37°C and white colonies were picked for analysis. Then the correct bacmids were transfect into insect cells to produce recombinant baculovirus and harvested 4 days later. SF9 cells were infected by the viruses and harvested 3 days later. Protein complexes were purified with Ni Sepharose 6 Fast Flow (GE Healthcare) and Flag agarose (Sigma) sequentially.

***In vitro* ubiquitination assays**

The plasmids and purified proteins of E1, E2 and Ub are gifts of Dr Wei Li from Institute of Zoology, CAS. GST-CCNE1 were expressed and purified from bacteria. The SPOP/CUL3/RBX1 complex was expressed with the baculovirus

expression system and purified from insect cells. In vitro ubiquitination assays were carried out by adding E1 (0.2 μg), 6His-UbcH5b (2 μg), GST-CCNE1 (5 μg), 6His-Ub (5 μg), SPOP-Cul3-RBX1 complex in ubiquitination buffer (20 mM Tris-HCl (pH 7.4), 10 mM ATP,10 mM MgCl2, 0.1 mM DTT) to a final volume of 50 μl. The reactions were incubated at 37°C for 1 h, and stopped with 1% SDS. The samples were heated at 95°C for 5 min and diluted 10-fold in lysis buffer (20 mM Tris-HCl (pH 7.4), 150 mM NaCl, 10% glycerol, 0.5% NP-40,1 mM DTT, 1 mM Aprotinin, 1mM Leupeptin) for immunoprecipitation or immunoblot analysis.

Cell proliferation assay

Cell proliferation was analyzed with MTT assay as previously described (Zhao et al., 2012). Briefly, cells were split at 1×10^3 per well in 96-well plates (n = 4). The cells were added with MTT (0.25 μg) in each well at the indicated time points before incubation for 4 h at 37 °C, the medium with the formazan sediment was dissolved in 50% DMF and 30% SDS (pH4.7). The absorption was read at 570 nm.

Colony formation assay

The cells were plated into a 12-well tissue culture plate (200 cells/well) and incubated at 37°C for 8 d. The resulting colonies were rinsed with PBS, then fixed with methanol for 10 min, and stained with methylthionine chloride. The colonies in each sample were photographed and counted by FlowJo software (n = 3).

Xenograft experiments of cancer cells

The five-week-old male BALB/C nude mice were purchased from Beijing HFK bioscience Co. Ltd. A RCC mouse model was established by injecting subcutaneously the animals with 1×10^7 769-P cells in the flank region. A prostate cancer mouse model was established by injecting subcutaneously the animals with 5×10^6 DU145 cells in the flank region. Tumor volumes were measured twice a week using calipers. Tumor volumes were derived as $V = 0.5\times\text{length}\times\text{width}^2$. After four weeks of injection, the tumors were harvested and weighed. All animal xenograft

experiments were performed following the university laboratory animal guidelines and were approved by the Animal Experimentations Ethics Committee of Wuhan University.

Supplemental Table S1 Information of primers and siRNAs

Assay	Gene Names	Forward	Reverse
RT-PCR	SPOP	GGTGCTACACACAGATCAAG	TAATGACTTCACCCATTTCC
	β-actin	CAGCACAATGAAGATCAAGA	GATCCACATCTGCTGGAAG
	CCNE1	GCCAGCCTTGGGACAATAATG	CTTGCACGTTGAGTTTGGGT
	LncRMRP	ACTCCAAAGTCCGCCAAGA	TGCGTAACTAGAGGGAGCTGAC
	SAMHD1	TCACAGGCGCATTACTGCC	GGATTTGAACCAATCGCTGGA
	RAMP1	CTGCCAGGAGGCTAACTACG	GCTCCCTGTAGCTCCTGATG
	APOE	GTTGCTGGTCACATTCCTGG	GCAGGTAATCCCAAAAGCGAC
	IFI27	TGCTCTCACCTCATCAGCAGT	CACAACTCCTCCAATCACAACT
	OAS1	TGTCCAAGGTGGTAAAGGGTG	CCGGCGATTTAACTGATCCTG
	TRIM14	TACATTACAGACGCCATTGGAC	GGGCTGGTTTTCAACAAGGT
	XAF1	GCTCCACGAGTCCTACTGTG	GTTCACTGCGACAGACATCTC
	LY6E	CAGCTCGCTGATGTGCTTCT	CAGACACAGTCACGCAGTAGT
	FBXW7	CTGAAGACAACCTTCTCTGG	TTTCTTCTCTTGATGTGCAA
	RhoBTB3	AGTAATTATTGCTGCTGTTGG	TAACACAGCTCCCTCTGTCT
siRNA	SPOP.1	AGAUCAAGGUAGUGAAAUU	
	SPOP.2	GCUUAAAGGUCAUGUGUGA	
	FBXW7	CCAUGCAAAGUCUCAGAAU	
	RHOBTB3	AGGAAAGUAUUUUGGAGGA	
	CCNE1	GCAAAAGGUUUCAGGGUAU	
shRNA	SPOP	AGATCAAGGTAGTGAAATT	
